# Elevation as a Grammatical and Semantic Category of Demonstratives

**DOI:** 10.3389/fpsyg.2020.01712

**Published:** 2020-07-30

**Authors:** Diana Forker

**Affiliations:** Department of Caucasus Studies, Friedrich-Schiller University of Jena, Jena, Germany

**Keywords:** elevation, vertical axis, space, deixis, time, demonstrative pronouns

## Abstract

In this paper I study semantic and pragmatic properties of elevational demonstratives by means of a typological investigation of 50 languages with elevational demonstratives from all across the globe. The four basic verticality values expressed by elevational demonstratives are UP, DOWN, LEVEL, and ACROSS. They can be ordered along the elevational hierarchy (UP/DOWN > LEVEL/ACROSS), which reflects cross-linguistic tendencies in the expression of these values by demonstratives. Elevational values are frequently co-expressed with distance-based meanings of demonstratives, and it is almost always distal demonstratives that express elevation, whereas medial or proximal demonstratives can lack elevational distinctions. This means that elevational demonstratives largely refer to areas outside the peripersonal sphere in a similar way as simple distal demonstratives. In the proximal domain, fine grained semantic distinctions such as those encoded by elevational demonstratives are superfluous since this domain is accessible to the interlocutors who in the default case of a normal conversation are located in close proximity to each other. I then discuss metaphorical extensions of elevational demonstratives to non-spatial uses such as temporal and social deixis. There are a few languages in which elevational demonstratives with the meaning UP express the temporal meaning future, whereas the DOWN demonstratives encode past. This finding is particularly interesting in view of the widely-debated use of Mandarin Chinese spatial terms ‘up’ for past events and ‘down’ for future events, which show the opposite metaphorical extension. I finally examine areal tendencies and potential correlations between elevational demonstratives and the geographical location of speech communities in mountainous areas such as the Himalayas, the Papuan Highlands and the Caucasus. I tentatively conclude that languages spoken in similar topographic environments do not tend to have similar systems of elevational demonstratives if they belong to different language families.

## Introduction

The expression of space in grammars of natural languages is ubiquitous and ‘spatial language’ has been investigated for decades within many different linguistic subdisciplines and by means of various approaches and frameworks. However, research on the spatial category of elevation is just at the beginning and typological studies are lacking so far. Elevation refers to the expression of a location of a figure with respect to the ground on the vertical axis. Many languages have words for ‘up’ and ‘down’ or ‘higher’ and ‘lower,’ but not all languages have this semantic distinction grammaticalized as part of certain closed class items, most notably demonstratives, which are the topic of this paper, but also spatial preverbs and case systems. Elevational meanings have repeatedly been grouped together with grammatical items that refer to salient landmarks (e.g., ‘seawards’/‘landwards,’ ‘upriver’/‘downriver’). Such systems have been called ‘environmental space deixis’ (Bickel, 1997), ‘spatial coordinate systems’ (Burenhult, 2008) or ‘topographical deixis’ (Post, 2011). From a number of surveys, we can conclude that demonstratives expressing elevational distinctions are cross-linguistically not extremely common but also not extremely rare, but we lack detailed comparative studies (e.g., Hyslop, 1993; Ebert, 1994; Diessel, 1999; Dixon, 2003; Post, 2011, 2017; Schapper, 2014; Aikhenvald, 2015; Breunesse, 2019).

In this paper, I concentrate on the semantic and pragmatic properties of elevational demonstratives, more specifically, adnominal, spatial adverbial, and pronominal demonstratives. This study therefore goes beyond general cross-linguistic studies of demonstratives, which devote only a few sentences to demonstratives with elevational meaning. It also goes beyond more specific surveys such as Post (2011, 2017) and Schapper (2014), which devote considerable space to elevationals, but focus on particular linguistic areas/languages families. I first lay out the conceptional and notional background for verticality and its relation to deixis, and describe morphological, syntactic and semantic properties of elevational demonstratives. I then propose the elevational hierarchy along which the basic elevational meaning categories can be ordered. Subsequently, I examine the relationship between elevational meaning and distance contrasts of demonstratives and further semantic extensions of elevationals to indicate cardinal directions, social hierarchies, and temporal meanings. The data for this paper mainly come from grammatical descriptions of some 50 languages with elevational demonstratives from a range of different language families across the globe.

## Conceptional and Notional Background

### Verticality Within the Domain of Spatial Language

**FIGURE 1:**
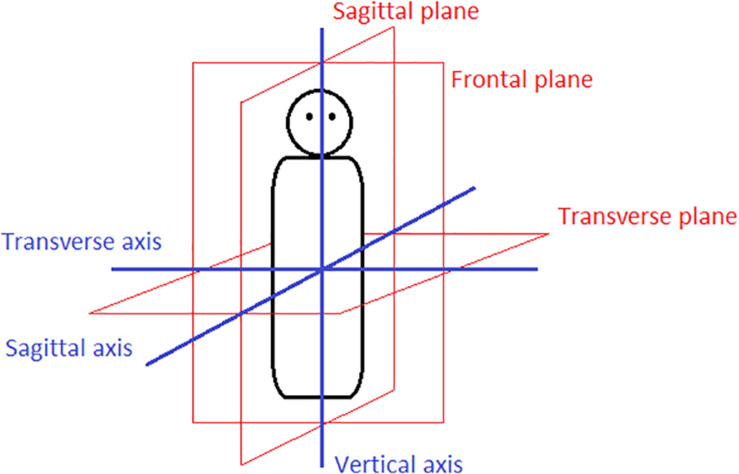
The three body planes and axes.

As said in the introduction, elevation refers to the expression of a location of a figure with respect to the ground on the vertical axis.^1^ The three axes and planes through the human body provide the ground for three pairs of (linguistic) concepts, namely UP/DOWN, BACK/FRONT, and LEFT/RIGHT (Figure 1). Languages have a plethora of linguistic means to express locations of objects along the vertical axis, e.g., A is *above/over* B, A is *higher* than B, A can be *upward*, *uphill*, *up the road* with respect to B. This exemplification of English prepositions, adjectives and adverbs is far from being exhaustive. However, many languages do not have words referring to the sagittal (back/front) or transverse (left/right) axes or do not employ them regularly and in the same fashion as familiar European languages (Levinson, 2003, p. 46). And what is more relevant for the topic of this paper, the vertical axis is the only of the three axes that is encoded by demonstratives. No language has been reported so far to have demonstratives for the other two axes.^2^

The vertical axis is special in comparison to the other two axes of the body (frontal and sagittal) (Figure 1) for one major reason: gravity normally determines what counts as *up* and *down*. The position of a figure *above* or *over* a ground object is usually defined by gravity and thus in most instances in practice absolute (see also Clark, 1973). Positions along the vertical axis cannot easily be rotated or reflected in contrast to positions on the back/front and the left/right axes (i.e., *front* becomes *back* or *left* becomes *right* through rotation or reflection). Locations *in front of X* or *left of X* are potentially ambiguous because they can depend on the relative viewpoint: By contrast, normally we unambiguously understand *above/over X* or *below/under X* if we know the position of X. Because of this (usual) unambiguity of locations along the vertical axis, the anchor point of an observer can shift without difficulty (we will see below what repercussions this has for elevational demonstratives). Levinson summarizes the distinctiveness of the vertical axis by stating that “the intrinsic (canonical position of objects), the relative (perception from an upright stance) and the absolute (as defined by the gravitational axis) tend to coincide” (Levinson, 2003, p. 75; see also Carlson-Radvansky and Irwin, 1993, p. 224 for the same observation). For a detailed explication of the concept of frames of reference in spatial language and its three basic types, intrinsic, absolute and relative, see Levinson (2003, pp. 24–61). Bender and Beller (2014, p. 348) provide useful graphic representations of the basic types and further subtypes.

The intrinsic frame of reference entails that the ground and the origin of the coordinate system that serves as anchoring point are identical and the spatial relation between the figure and the ground is binary. In an absolute frame of reference, there is also a binary relation, but this time between the ground and independently given salient geographical landmarks or cardinal directions that serve as anchoring points (e.g., *north of X*). By contrast, in a relative frame of reference there is a ternary relation because in addition to the figure and the ground (relatum or ground object) there is an anchoring point (=the origin of the coordinate system).

**FIGURE 2:**
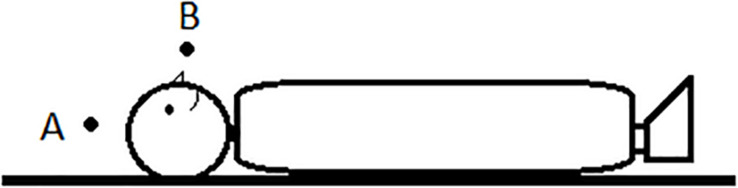
Location ‘over the head.’

As was just said, the vertical axis is special because of its natural grounding in gravity. However, we can ‘escape gravity’ in the sense that we can change the frame of reference from absolute to intrinsic or relative. Figure 2 shows a person stretched out on the ground. The description of object B as *over the head* entails a relative (to an external upright observer) or absolute frame of reference as determined by gravity.^3^ We make use of an intrinsic frame of reference when we refer to object A, which is located at the same elevation of the head of the person and aligned with it along the same horizontal axis, as *over the head*. However, in none of the languages in my sample I encountered examples illustrating an elevational demonstrative used with an intrinsic frame of reference (i.e., DEM.UP A). From a logical point of view there is no reason to exclude such usages, but their actual existence has yet to be proven by future research.^4^

The peculiarity of the vertical axis has also been examined in psychology. Vertical spatial relations among objects remain largely constant with respect to a moving observer whereas on the transverse (i.e., horizontal) plane spatial relations change more frequently. Therefore, human beings are faster at retrieving the names of objects located along the vertical axis than along the other two axes (Bryant et al., 1992).

With respect to the topic of this paper the category of deixis comes into play because the items examined are either categorized as demonstratives themselves or as parts (bound roots, affixes, or clitics) of demonstratives. Following Diessel (1999, p. 2; see also Dixon, 2003), demonstratives are deictic expressions serving specific syntactic and pragmatic functions. Commonly distinguished categories of deixis are person, place, time, discourse, and social deixis (Diessel, 2012, p. 2414), and demonstratives usually express place deixis/spatial deixis (Diessel, 1999, p. 36). The term ‘spatial deixis’ refers to the localization of a figure relative to a ground (object) in terms of (radial) distance categories by means of language (e.g., *here* vs. *there*), or in combination with a pointing gesture (Levinson, 2003, p. 65). The deictic center is usually egocentric, i.e., the speaker’s location serves as the ground, but can also shift depending on the speech situation. According to the survey in Diessel (2013), which included 234 languages, demonstratives are distance-neutral or express up to five distance contrasts (i.e., five positions that differ in terms of distance from the deictic center). In purely distance-based systems, the deictic center is the speaker (i.e., egocentric system) and thus identical for all demonstratives. Among the languages with a three-way distance contrast (88 languages in Diessel, 2013), around one third are so-called person-oriented or person-based systems. This means that one of the three demonstratives expresses proximity to the hearer, and therefore the deictic center is not the speaker, but the hearer.

I will discuss the interaction of deixis with elevation in the Section “The vertical dimension and its relation to deixis” after having described in more detail elevational meanings.

## Materials and Methods

### Language Sample

For this paper I surveyed elevational demonstratives in 50 languages from 20 language families plus one isolate. My sample is a convenience sample because elevational demonstratives are not particularly frequent in the world’s languages. Many of the languages have been identified through the works by Diessel (1999); Post (2011, 2017); Sarvasy (2014), and Breunesse (2019). In addition, an unpublished database by Killian (unpublished), which contains data on demonstrative systems in around 1,100 of the world’s languages, served as a major reference. According to Killian, the database is not completely unbiased, but it covers all areas of the world and more than half of the world’s language families.

The elevational demonstrative systems of the 50 surveyed languages have been coded for a number of formal and semantic properties. The list of languages, schematic overviews of the elevational demonstrative systems together with genealogical and geographical information on the area where the languages are spoken and references are given in the Supplementary Appendix Table A12. Language families and subbranches in which elevational demonstratives are attested for many languages are East Caucasian languages, Eskimo-Aleut languages, Sino-Tibetan (in particular Bodic languages, Kiranti languages, Macro-Tani), Timor-Alor-Pantar languages, Nuclear Trans New Guinea, and Omotic languages.

### Morphological and Syntactic Features

In general, demonstratives can be bound and unbound forms, whereby the bound forms are normally clitics and not affixes (Diessel, 1999, p. 22–25). They can be morphologically simple and complex. The same is true for the subclass of elevational demonstratives, but with a further complication because elevation constitutes an additional semantic component on top of the basic demonstrative meaning (which is distance-based and/or person-based). This additional semantic component is either not expressed by a separate morpheme and then part of the basic demonstrative stem, or it is expressed by a separate morpheme. For this study, morphemes were considered elevational demonstratives if they combine with a demonstrative stem in a single lexical item, or appear to express both demonstrative and elevational functions.^5^ In other words, elevational demonstratives are often morphologically and always semantically complex expressions that constitute single word forms. Based on these considerations, the items under investigation can be divided into three basic types:^6^

(i) Co-expression of elevational and demonstrative meaning in a single morpheme.
(ii) Obligatory co-occurrence of demonstrative morphemes with elevational morphemes in a single word-form.
  (a) No occurrence of elevational morphemes outside these forms.
  (b) Occurrence of elevational morphemes outside these forms.
(iii) Optional co-occurrence of demonstrative morphemes with elevational morphemes in a single word-form.
  (a) No occurrence of elevational morphemes outside these forms.
  (b) Occurrence of elevational morphemes outside these forms.

The elevational morphemes that obligatorily or optionally co-occur with demonstrative morphemes are bound roots, affixes or clitics. Based on the descriptions it is not always possible to distinguish between the subtypes (ii) and (iii) because not all grammars explicitly state whether the elevational morpheme also occurs in some other parts of speech (e.g., as preverb or spatial case affix).

Co-expression of elevational and basic deictic demonstrative meaning at the synchronic level occurs in Muna (Table 1), Daga (Table 9), Yakkha (Table 11), Iaai (32), (33), Jahai, Abui, Tidore, Sougb, Tulil, Hatam, Fore, Usan, Yale, Dadibi, and Zayse. Table 1 shows the demonstrative system of Muna (Malayo-Polynesian, Sulawesi). Out of six demonstrative forms (with anaphoric and deictic variants), only one (*tatu*) co-expresses the elevational meaning UP and the deictic meaning distal. Only when occurring in opposition with *tatu*, the neutral distal demonstrative *watu* can also mean DOWN or LEVEL.

**TABLE 1:** The structure of Muna demonstratives (van den Berg, 1989/2013, p. 89).

	Anaphoric	Deictic
S-proximal	*ini*	*a-ini*
H-proximal	*itu*	*a-itu*
Away from S, H, but nearby	*maitu*	*a-maitu*
Far (neutral)	*watu*	*a-watu*
Far (high) [UP]	*tatu*	*a-tatu*
Not visible, audible, unspecified for time	*nagha*	*a-nagha*

Eipo (Mek, Eastern Highlands of Papua New Guinea) has two subsets of demonstratives (Table 2). The elevational values in both subsets are obligatorily co-expressed with the deictic meaning DISTAL. The second subset, which contains the intensifier *d*-, expresses additional distance or contrast.

**TABLE 2:** The structure of Eipo demonstratives (Heeschen, 1982, pp. 84–86; Heeschen, 1998, p. 143).

		+ Additional distance
Proximal (‘here’)	*a-*	*d-a-*
Distal high (‘up there, above’) [UP]	*ei-*	*d-ei-*
Distal down (‘down there’) [DOWN]	*ou-, u-*	*d-ou-*
Distal across (‘across there’) [ACROSS]	*or-, er-*	*d-or-*

There are a number of languages such as Baskeet, Yupno, Makalero (Table 3), and Khasi, which obligatorily require further morphology to be added to the elevational demonstrative. This can be gender marking as in the pronominal demonstratives in Baskeet (8) or in Khasi. Or it can be derivational suffixes for the formation of demonstrative pronouns, adverbs or verbs as in Yupno and Makalero, and Khasi adverbial demonstratives. The elevational demonstrative morphemes themselves cannot be clearly separated further and no unambiguous part with purely elevational meaning can be identified. For example, in Makalero (Alor-Pantar, East Timor) nominal and verbal demonstratives are derived from the same bound roots by means of the nominalizer -*r*- and the verbalizer (glottal stop; Table 3).

**TABLE 3:** The demonstrative system of Makalero (Huber, 2011, p. 232).

	Meaning	Nominal demonstratives	Deictic verbs
Same	Proximal to speaker	*ere*	*e’*
level	Proximal to hearer	*uere*	*ue’*
	Distal from speaker and hearer	*umere*	*ume’*
	Higher elevation [UP]	*udere*	*ude’*
	Lower elevation [DOWN]	*ufere*	*ufe’*

In a number of languages, the elevational demonstratives are clearly diachronically complex, but synchronically the elevational part cannot be separated or is not treated as a bound root, affix, or clitic. Languages belonging to this type are Sanzhi Dargwa, Hua (Yagaria dialect), Central Alaskan Yupik, Kurtöp (9), (22), and Galo (19).

Languages with morphologically complex elevational demonstratives in which the elevational meaning is expressed by bound roots or affixes and regularly combines with demonstrative stems are Blagar, Tauya, Tanacross (Table 4), Koyukon, Andi (Table 5), Manambu (Table 8), Ngiyambaa (7), and Dyirbal (10). For example, demonstratives in the Athabaskan language Tanacross (Alaska) morphologically and semantically combine deictic meaning (distality) with specific topographic and elevational morphemes. The topographic and elevational morphemes express also directional and locational meanings (e.g., allative).

**TABLE 4:**
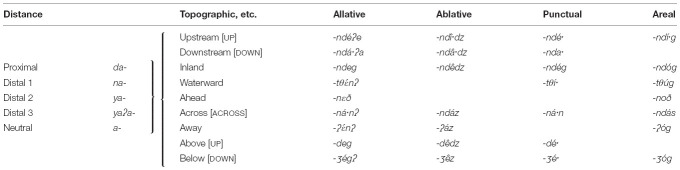
The demonstrative system of Tanacross (Holton, 2019).

**TABLE 5:** The structure of adnominal and pronominal demonstratives in Andi (Verhees, 2019).

Stem: *hV* distance/person-based deixis	Emphasis	Elevation	Gender, (number)
Proximal *ho-*	-*n(V)*	Same level [LEVEL] -*dV*	Singular human male -*w*
Medial *he*-		Lower [DOWN] -*gV*	Singular human female -*j*
Distal *hu*-		Higher [UP] -*ɬV*	Singular neuter 1 -*b*
Distal *hi*-			Singular neuter 2 -*r*

The demonstratives in the East Caucasian language Andi (Zilo dialect, Caucasus, Russia) are particularly transparent and consist of stems that express distance- and person-based deixis, followed by a range of further optional suffixes such as an emphatic marker, the elevational morphemes and gender suffixes (and/or oblique stem markers and case suffixes not displayed in Table 5).

The classification introduced at the beginning of this section makes a distinction between (a) and (b) subtypes, whereby the (a) subtypes refer to elevational morphemes that only combine with demonstrative morphemes, whereas the (b) subtypes of elevational morphemes also occur outside the demonstrative systems. In Andi and Manambu, the elevational markers are only used with the deictic demonstratives and thus belong to the (a) subtype. By contrast, in Dyirbal they can also be added to verbs to form verbs of motion (Dixon, 1972, pp. 57, 322), and thus Dyirbal belongs to the (b) subtype. Similarly, in Eipo, Sougb, Nêlêmwa-Nixumwak, and Abui^7^ deictic motion verbs can attach the elevationals.

However, this cross-categorical formal flexibility is not the rule. There are a few languages in my sample that have specialized motion verbs referring to upward or downward movement, but the elevational markers that those verbs contain are historically unrelated to the elevational demonstratives (Galo, Sanzhi Dargwa, Yupno, and Bantawa).

For the (b) subtypes, the question can be asked what the nature of the elevational morpheme is, in particular, whether they are themselves deictic or non-deictic. However, for this paper the answer to that question is largely irrelevant, because I am only interested in the combined forms, i.e., the co-occurrence of demonstratives and elevational morphemes. This touches upon a problem I encountered during this study. I had to rely on the often implicit assumptions of the linguists whose descriptions I consulted that the items classified as ‘elevational demonstratives’ represent single lexical units. In languages such as Manambu, Sougb, or Nêlêmwa-Nixumwak, in which the morphemes with the elevational semantics can be readily identified and are sometimes also used with lexical items other than demonstratives (e.g., verbs), the elevationals resemble English non-deictic expressions such as *up*. English *up* can co-occur with adverbial demonstratives (*up there*) and verbs (*climb up*). However, no linguist has ever claimed that English has an elevational demonstrative although such a claim would perhaps be imaginable if we wrote *up-there* or *upthere* instead of *up there*. This means that among the languages studied for this paper there might be languages that are actually not extremely different from English, but for which the author of the grammar has reasons to assume that a morphologically and semantically complex expression translates with, e.g., *up-there* or *upthere*, constitutes a single lexical item.

Most of the elevational demonstratives take further optional or obligatory derivational and/or inflectional suffixes (most commonly gender, number, case, nominalizers or adverbializers). They are part of paradigms or subparadigms that consist of three (Andi) to five (Makalero, Manambu, and Buru) items on average, but more than seven members are not exceptional (Daga). For instance, Tanacross has nine items (Table 4), and Movima even has 14 basic demonstratives occurring in paradigmatic relationship. See Diessel (1999, pp. 32–33) for general morphological properties of demonstratives, which also apply to elevational demonstratives.

Diessel (1999, p. 57) distinguishes four syntactic contexts in which demonstratives occur. These contexts are (i) pronominal use, (ii) adnominal use (i.e., as determiner), (iii) spatial and manner adverbial use, and (iv) identificational use in copula and non-verbal clauses. The identificational context of use has been and/or is also called ‘predicative’ use (e.g., in the first typological paper on this topic written by Killian, unpublished.). As stated in the introduction, I focus on adnominal, spatial adverbial, and pronominal elevational demonstratives. Because other forms need further research they will only be mentioned in passing. Examples (1)–(4) illustrate all four contexts.


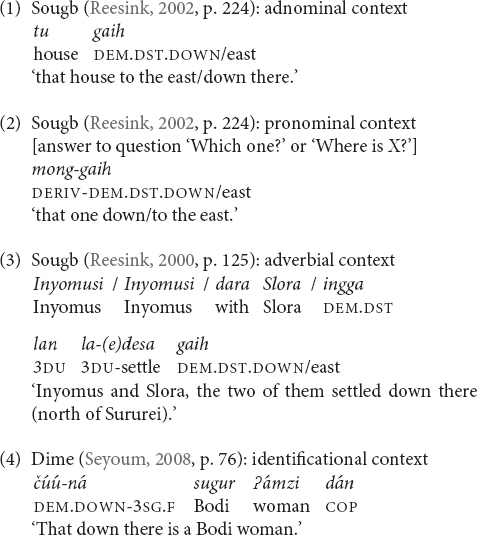


The last context (iv) has several subtypes (presentative, identifier, localizer, and copular demonstratives, see Killian, unpublished for the full typology, explanations and examples). Copular demonstratives are cross-linguistically rare (Killian, unpublished; see also Guérin, 2015). Among the languages in my sample Blagar, Makalero, and Tidore have elevational demonstrative verbs with the meanings ‘be here/there up/down’ that exhibit predicative use:


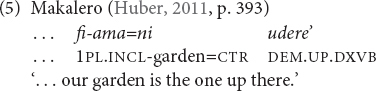


The four basic contexts are attested to various extents for elevational demonstratives. In Maale, only the adverbial use is found. The adverbial context can be considered the minimal context of use probably attested for all languages in my sample. The adverbial use normally refers to the occurrence of elevational demonstratives in the function of spatial adverbs (3), (24). Makalero and Tidore do not have genuine elevational adverbial demonstratives, and the adverbial function is fulfilled by demonstrative verbs (5). Blagar, Galo (26) and all East Caucasian languages in my sample (Avar, Lak, Andi, and Sanzhi Dargwa) have not only spatial elevational demonstratives, but also a further class of elevational demonstratives that function as manner adverbs, e.g., Blagar *do-laŋ* (up.there-as) ‘like that/those up there (not necessarily visible)’ (Steinhauer, 2014, p. 159).

All languages expect for Tanacross and Maale employ elevational demonstratives in the adnominal context, and this is therefore the second most commonly attested type of usage. In Usan and Eipo, elevational demonstratives can be used as modifiers within a noun phrase (i.e., adnominal use), but not in the syntactic function of determiners. Instead, they co-occur with determiners.

The degree to which the syntactic contexts are expressed by specialized, formally distinct elevational demonstratives varies. I did not come across any language that always distinguishes all four types formally. Nungon makes formal distinctions between the first three syntactic contexts (Sarvasy, 2014, pp. 404–419). Sanzhi Dargwa and other East Caucasian languages formally distinguish elevational demonstrative adverbials (with spatial and manner semantics) from nominal demonstratives by means of derivational suffixes, and also has a separate class of copular demonstratives. Nominal demonstratives can be used adnominally or pronominally in Sanzhi, but they are only case-marked in the latter use (and thus formally distinct). The elevational demonstratives of Baskeet, Tauya, Galo, and Kurtöp seem to pattern alike. In Sougb, the pronominal and/or identificational use requires additional morphology (2), but adnominal and adverbial uses are identically and unmarked (1), (3). In Yakkha, the unmarked forms function as adverbials (12), and the adnominal forms are derived (15). Yale does not formally distinguish between adnominal and adverbial elevational demonstratives (and the author of the grammar does not explicitly mention a pronominal or identificational use).

### The Basic Semantic Distinctions of Elevational Demonstratives

Semantically, elevational demonstratives are deictic expressions that also convey elevational or verticality distinctions. Following Schapper (2014), I distinguish four basic concepts for verticality values and will employ them in the glosses of examples in order to facilitate understanding and comparison (6) (even though individual authors may use alternative terms, e.g., *higher*, *upward*, or *above* instead of UP).^8^ The term LEVEL includes a more specific term ACROSS:


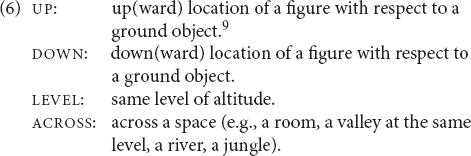


I further adopt and simplify the classification of Burenhult (2008) and differentiate between two basic types of elevational systems:

(i) General elevational demonstrative systems:
  • The location is determined according to an imagined vertical (longitudinal) axis that runs through the ground (e.g., human body).
(ii) Topographic elevational demonstrative systems:
  • The location is determined with respect to the geophysical environment.

The first type (‘general’) corresponds to Burenhult’s ‘verticality proper’ and ‘global elevation,’ and the second term (‘topographic’) to his ‘geophysical elevation.’ General elevationals are used in accordance with the gravitational axis. They can have very local meanings, which means that they can be applied, for instance, to refer to positions close to the speaker, inside a room or in the immediate environment (7), (8) but they are also used to denote locations in the geophysical environment (9).


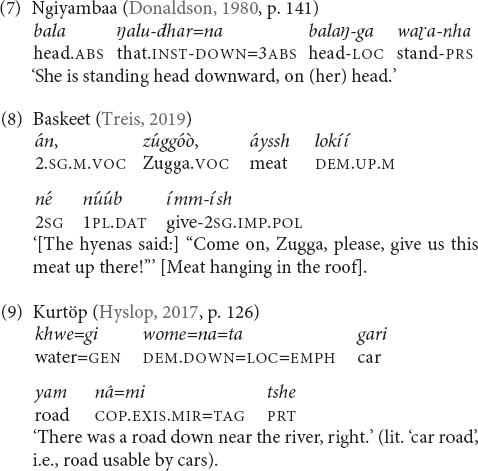


Genuine topographic elevationals refer on the basis of the geophysical environment. There are two types of landmarks outside and generally further away from the speaker that naturally expand along the vertical dimension, namely topographical contour (i.e., mountains including hills or large rocks) and hydrological contour (i.e., rivers and creeks).^10^ The vertical dimension of rivers might not be obvious at first glance. But what connects rivers with what was said before about the vertical axis is the fact that gravity causes the flow of the water in a certain direction and the direction is absolute and independent of an anchoring point. I did not find any other types of landmarks defining topographic elevational demonstratives.

Topographic elevational demonstratives basically mean something like ‘uphill’/‘downhill,’ ‘upriver’/‘downriver’ and the like. For instance, Dyirbal has an elaborated set of twelve so-called ‘spatial indicators’ that are added to demonstratives or other noun markers and express topographic elevation, e.g., ‘downhill,’ ‘uphill,’ ‘downriver,’ ‘upriver,’ and ‘across river’ (Dixon, 1972, p. 48; Dixon, 2003, p. 98). Dixon further adds that ‘river’ is the more specific meaning and the other terms translated by ‘hill’ rather mean ‘not river’ and can also refer to locations such as cliffs or trees. The topographic elevationals can be followed by another marker from a smaller set that contains only three items that encode general elevation and the meaning ‘out in front’ but also seem to have some additional meanings that are not explicitly discussed in the grammar (Dixon, 1972, p. 48).


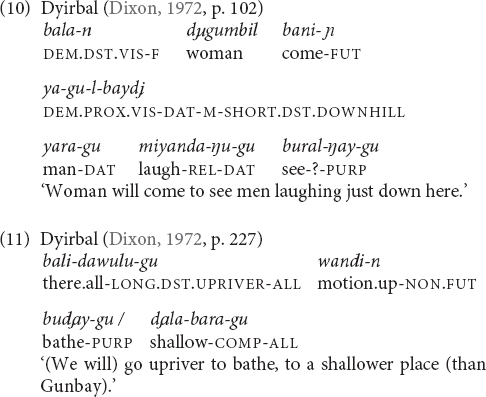


The distinction between general and topographic elevational demonstratives applies not just to the UP and DOWN meanings but also to LEVEL and ACROSS. For instance, terms that express ACROSS can be topographic and refer to locations across a valley at the same altitude of the opposite mountain as in Yakkha (12), or across the river as in Tanacross (13). They can also be general as in Usan and applied in the local domain (14).


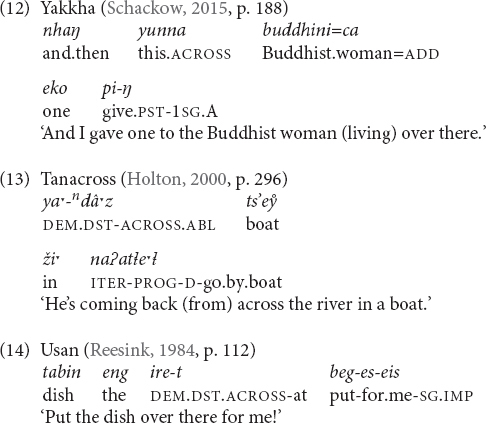


Levinson (2018, pp. 27, 35) states that topographic demonstratives make use of an absolute frame of reference because the referent is located “on a notional gradient (upriver/downriver and uphill/downhill) which actually delivers an angle on the horizontal.” He adds that such local landmarks do not have the same abstract properties as cardinal directions (Levinson, 2003, p. 90). This hints at one major problem concerning research on elevational demonstratives. Several languages have been claimed to possess topographic elevationals that employ an absolute frame of reference, but these claims are normally not proven by a comprehensive argumentation and detailed data. I suspect that these claims are probably sometimes wrong or at least misleading because, first, the authors do not provide unambiguous evidence that the relevant items refer on the basis of the geophysical environment and not simply to the vertical dimension. Second, the descriptions lack a solid proof of the absolute frame of reference as opposed to the relative or intrinsic frame.

**FIGURE 3:**
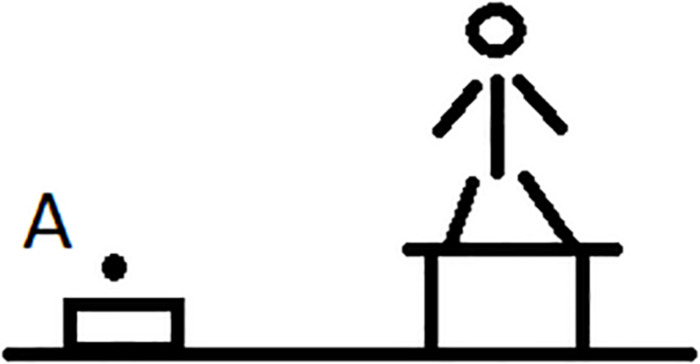
Elevation and the relative frame of reference.

In order to prove that an elevational demonstrative really makes use of an absolute frame of reference one has to explicate the coordinate system that serves as the observer-independent anchoring point in a similar way as cardinal directions. Above I explained that gravity is the natural source for the direction of elevationals and thus for the determination of what counts as UP and what as DOWN independently of an observer or an intrinsic orientation of the ground. This type of absolute frame of reference is also entailed in many usages of adverbs or adjectives such as English *up* vs. *down* or *high* vs. *low* (Clark, 1973), but these items can also be used with a relative frame of reference or an intrinsic frame of reference. What is thus needed when describing elevational demonstratives is to test if they can also refer to the position A in Figure 2 (intrinsic frame), or relative to an anchor point that is distinct from the observer, e.g., to object A in Figure 3, or if such usages are always excluded. Only in the latter case the meaning would truly entail an absolute frame.

**FIGURE 4:**
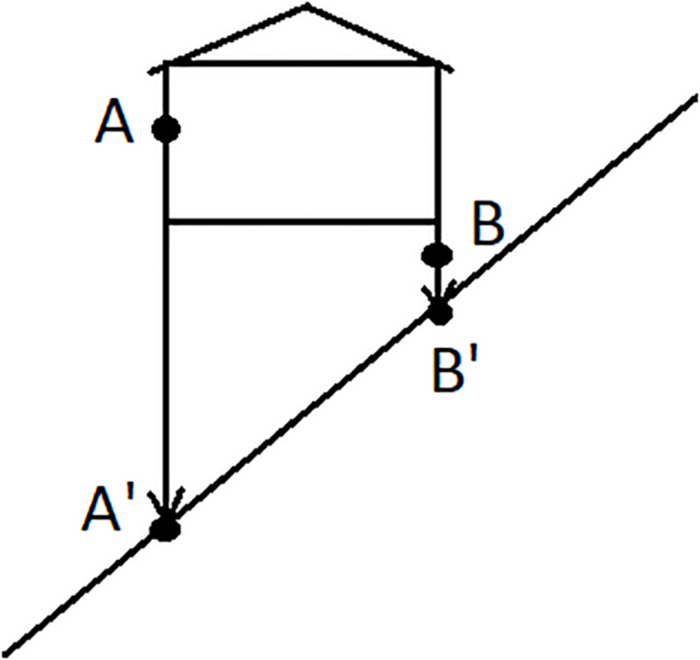
Topographic elevationals.

Second, a simple translation of a demonstrative as ‘uphill’ is not a proof for its topographic meaning with an absolute frame of reference. In particular, it is not sufficient if the demonstrative only occurs in example sentences that refer to people, animals, and other relatively big objects such as trees or houses and their location in the outside geophysical environment. If it is really a mountain or river that serves as the absolute landmark, then in a situation such as the one depicted in Figure 4, location A is ‘downhill’ and location B ‘uphill’ even though on a general vertical axis A is located further away from the ground and thus higher than B. In topographic systems the locations of the points A and B are projected on the ground and the positions of A’ and B’ determine the use of the appropriate demonstratives.

An example in point comes from Yakkha, which has two types of topographic elevational adverbials of which elevational demonstratives are formed (see Table 11 for the first type). In Yakkha, a spider can be referred to as being on the ‘downhill’ side of the speaker, even if it is located on the same elevation level as the speaker and thus factually not lower than the deictic center (Schackow, 2015, pp. 188–189).

Another important factor to keep in mind when investigating the meaning of elevational demonstratives is scale or domain of use. We have to distinguish at least three domains (which obviously form a continuum and therefore lack clear borders):

(i) The local domain: the minimal local scale is the peripersonal sphere, but it extends to the area inside a house or its immediate surrounding; locations within this area are often visible.
(ii) The local larger, but delimited environment, e.g., a village, a valley, or an island; locations within this area can be visible or not.
(iii) The global scale, e.g., locations on other continents that are never visible from the location of the speaker.

Since general elevationals can normally be used both in the local domain and in the larger domain (and sometimes even at the global scale), they have to be carefully distinguished from topographic elevationals that are projected into the minimal local domain.

Metaphoric usage extensions, projections onto the horizontal plane and conventionalized uses can create problems for the correct categorization of elevational demonstratives as general or topographic because they might obscure the basic elevational meanings. For example, Sanzhi Dargwa has a general elevational system clearly based on an abstract vertical axis (Forker, 2019). However, at the scale of the main modern settlement, which is located in the lowland coastal area close to the Caspian Sea with virtually no differences in height, there is an ‘upper’ part of the village located closer to the hills and a ‘lower’ part located closer to the sea coast. When talking about inhabitants of the village, a person might conventionally be referred to by an UP or DOWN demonstrative based on the permanent location of her house within the village, which is mentally divided into an upper part and a lower part, and not on the location of that person with respect to the speaker or another spatial anchoring.

Elevational demonstratives that are characterized as ‘topographic’ in grammars can be used at the local scale such as within a house or close by a house or, with respect to a tree. For instance, Tanacross and other Northern Dene languages have genuine topographic elevational systems (in addition to the general elevational demonstratives) that conventionally extend to the micro level. This means that within a house there are four directions/locations, namely ‘upstream,’ ‘downstream,’ ‘inland,’ and ‘across’ because traditionally houses have been built with the door toward the water (Holton, 2000, p. 298). Therefore, an object is, for example, located ‘upriver’ when its location is referred to with topographic demonstratives. The division of the areas within a house are even used within modern houses that do not always face the water. In this language, ‘uphill’ location is at the same time away from the river, and ‘upriver’ (‘upstream’) is along the river and thus orthogonal to ‘uphill’. This means that in terms of cardinal directions and gravity (i.e., location above sea level) ‘uphill’ and ‘upstream’ differ (Gary Holton, p.c.).

Similarly, by means of the second topographic system of Yakkha the ‘uphill’ and ‘downhill’ elevationals can be mapped onto the human body and teeth are then referred to as uphill, i.e., ‘upper teeth’ and downhill ‘lower teeth’ irrespectively of their actual position (even when a person is not in the canonical upright position).


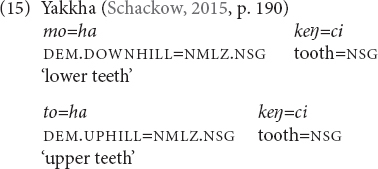


The two systems (general and topographic) as portrayed so far are idealized prototypes. Based on the descriptions that I consulted it is not always possible to determine if an elevational system falls into the one or the other category. In addition, it seems that there are systems that cannot be categorized as truly belonging to the one or to the other type, or should be analyzed as combining both types. For instance, the elevationals of Galo are translated as given in (16) (Post, 2007, pp. 349–350).


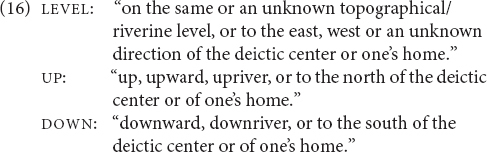


They are used at the local scale (17), the larger local scale (18) and the global scale (26). Only when the referent is potentially visible (i.e., within the minimal local and larger local domain) the relevant items encode elevational and riverine meanings.


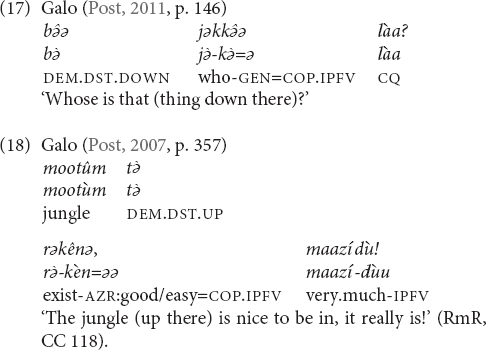


When the referent is not potentially visible and also not located on the path of a nearby river, but is separate from the speaker by at least a mountain range (i.e., global scale), then the same items function as labels for cardinal directions (19), and elevational differences are ignored. For instance, the speaker who uttered (19) is located in a village at around 100 m above sea level and Itanagar, where he would like to go, is situated at around 440 m and thus higher, and to the south but not visible from his village. In such a context, the anchor point can be the actual location of the speaker, or her/his home village can serve as conventionalized anchor point (similar to the conventionalized use of Sanzhi Dargwa demonstratives mentioned above). For unknown locations, the LEVEL items can be used as default demonstratives.


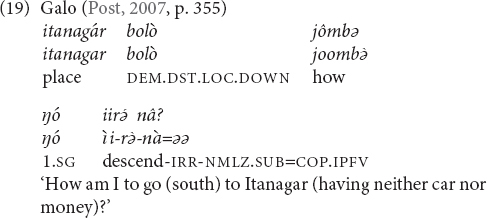


Four languages in my sample have two separate sets of elevational demonstratives, one set of general and another set of topographic elevationals (Dyirbal, Tanacross, Cora, and Buru, Table 6). In Buru (Malayo-Polynesia, Moluccas of Indonesia) topographic elevationals express three elevational values (UP, DOWN, and ACROSS) and general elevational morphemes only two (UP and DOWN) (Table 6). Grimes (1991, p. 170) does not provide a precise definition for the term ‘emic,’ but writes that the concept ‘away from an emic center’ as it is expressed by the topographic demonstrative *lawe* in Buru indicates ‘energy directed away from the actor.’ It is possible though not unambiguously clear from the description that this formulation can be translated into ‘away from the speaker.’

**TABLE 6:** The structure of Buru demonstratives (Grimes, 1991, p. 168).

Distance and definiteness		Topographic and general	
*naa*	Definite proximal	*pao*	Down, downward [DOWN]
*dii*	Definite distal (non-proximal)	*lawe*	Downstream/away from emic center/far [DOWN]
*saa*	Indefinite (specific or non-specific)	*saka*	Up, upward [UP]
		*dae*	Upstream/toward emic center [UP]
		*aki*	Across (stream, valley, ridge) [ACROSS]


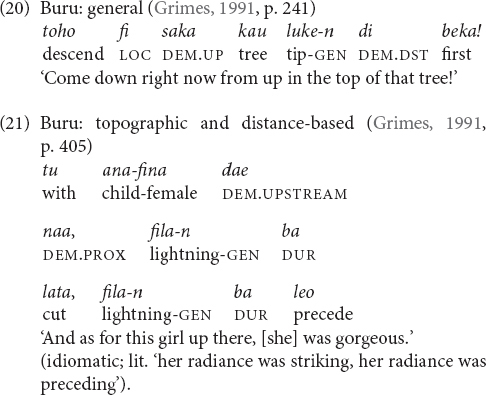


### The Vertical Dimension and Its Relation to Deixis

As stated in Section “Verticality Within the Domain of Spatial Language” above, demonstratives are deictic and express distance-based meanings with the speaker (ego) as deictic center or person-based meanings that additionally consider the position of the hearer. Verticality is not inherently deictic because the ground or anchoring point is not exclusively the speaker (Fillmore, 1982, pp. 39, 51; Diessel, 2012, p. 2,421). Nevertheless, terms expressing verticality can be relational and they can be used with relation to the speaker, which then may lead to the impression that the verticality component in elevational demonstratives is, by itself, deictic.^11^ For instance, Kurtöp elevational demonstratives have been glossed as deictic with the speaker as deictic center. However, in (22) the UP-demonstrative occurs together with the hearsay evidential, which means that the speaker has acquired her/his information from the speech of others. This is a clear indication that the speaker cannot be the deictic center that serves as the point of anchoring for the location of the woman. The location of the woman is rather described as being higher than before after she had climbed up to the top of the roof.


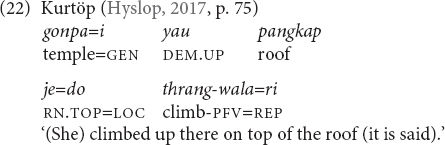


If elevationals were deictic by themselves, they would be ego-centered or only allow for shifting the deictic center to another speech act participant. But several descriptions explicitly mention that the anchor point serving as the ground (=deictic center) for elevational demonstratives can easily shift, e.g., in a story it shifts to a protagonist or to another salient inanimate anchor point [Tulil as analyzed by Meng (2018) and Ma Manda as examined in Pennington (2016)].^12^

Furthermore, as illustrated by means of Figures 2, 3 and in the discussion of the preceding section, when studying elevational morphemes it is necessary to examine whether they allow not only for the absolute frame of reference but if intrinsic and relative interpretations are also available. As I already explained, it is sound to expect the absolute use to be the default such that the interpretation of ‘down there’ in (17) is normally understood in relation to the position of the speaker and not some other ground object because of gravity. However, since we know that other elevational terms such as ABOVE or BELOW can, in principle, be employed within intrinsic and relative frames of reference, it is desirable in future research on elevational demonstratives to systematically test if there are any elevational demonstratives that can also be used in that way.

## Results

### The Elevational Hierarchy

In (6), I introduced the basic terms for verticality values. These values can be ordered along the elevational hierarchy that reflects cross-linguistic frequency of occurrence (23):


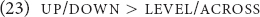


Elevational demonstratives with the meanings UP and DOWN are more commonly found than those with the meanings LEVEL or ACROSS (Table 7). All languages with LEVEL or ACROSS elevationals also have DOWN and UP elevationals. A minimal system of elevational demonstratives consists of one item for UP or one item for DOWN, but far more common is to have one term for each of the values UP and DOWN. So far, I did not find any language with both LEVEL and ACROSS elevational demonstratives, so these two values seem to exclude each other (although semantically ACROSS can be considered a sub-category of LEVEL). The more specific value ACROSS (8 languages) occurs only around half as often as LEVEL (19 languages).

**TABLE 7:** The frequency of elevational systems classified according to basic elevational meanings^1^.

	DOWN	UP	UP + DOWN	UP + DOWN + LEVEL	UP + DOWN + ACROSS
# of languages	1	2	21	19	8

*^1^I include systems that have items with elevational meanings for which I am not entirely sure that they fit the definitions. These items are given in parenthesis in Supplementary Appendix Table A12, e.g., U/D/(L). A few languages have more than one set, therefore the total for the number of languages in Table 5 is higher than the number of languages surveyed. Don Killian (p.c.) pointed out three more languages that are relevant for the topic of this paper, but not included in the Appendix and in the table: Moskona (Gravelle, 2010, pp. 199–200) that has UP (‘upward’) and ACROSS, which seems to be a very rare combination because it omits DOWN. Tepehuan (Willett, 1991, p. 92) has a separate term for UP (‘higher’) and combines DOWN (‘lower’) and LEVEL in one term, and Edolo (Gossner, 1994, pp. 85–87) that has two items for DOWN (‘that below,’ ‘that far below’), but just one for UP and one for LEVEL.*

Eipo and Andi and have all three types of demonstratives (Tables 2, 5); Manambu has UP and DOWN (Table 8), and Muna has just UP (Table 1). Hatam has even two terms for UP (*nyo* ‘sloping up’, *hu* ‘vertically up’), but only one for DOWN (*mu*) (Reesink, 1999, pp. 60–61).

**TABLE 8:** Structure of Manambu demonstratives (Aikhenvald, 2015).

Stem	Suffixes	Topographic and general
S-proximal *kə*-	Feminine singular *-l*	Up [UP] -*wur*
H-proximal *wa*-	Masculine singular -*d*	Down [DOWN] -*d(a)*
Distal *a*-	Dual -*bər*	Across [ACROSS] -*aki*
	Plural -*di*	Outwards -*aku*
	Current relevance -*na*	Off-river -*wula*

### Elevation, Distance and the Sagittal Axis

In their demonstrative systems, languages repeatedly combine elevation with distance. This means that the values DISTAL and PROXIMAL (and also MEDIAL for those languages that make a ternary distinction) are either obligatorily co-expressed or optionally combined with elevational items if the elevationals are morphemes that are formally independent of the distance-based deictics. There are languages in which all distance-based deictics can be combined with all elevationals. For example, in Manambu (Ndu, Sepik, Papua New Guinea), three person-based deictic stems take gender, number and the current relevance suffix, followed by the topographic and general elevational morphemes (Table 8).

In many other languages there are some restrictions. Daga (Papuan), for instance, has a particularly rich system with 14 demonstratives, of which two are merely person-based, eight co-express three distance-based meanings (CLOSE, DISTAL, and FAR DISTAL) with the elevational values UP, DOWN, and LEVEL, and four more encode only elevational meanings (Table 9). Yupno combines MEDIAL and DISTAL but not PROXIMAL with elevationals (Cooperrider et al., 2017, p. 771).

**TABLE 9:** The structure of Daga demonstratives (Murane, 1974, p. 38).

Close to speaker	*ma*	Close to hearer	*ame*
Close higher [UP]	*uta*	Close lower [DOWN]	*ita*
Distal higher [UP]	*utu*	Distal lower [DOWN]	*isi*
Far distal higher [UP]	*use*	Far distal lower [DOWN]	*ise*
Same level [LEVEL]	*ata*	Far distal same level [LEVEL]	*ase*
Overhead [UP]	*oea*	Underneath [DOWN]	*ea*
Up, high [UP]	*ao*	Down, low [DOWN]	*ae*

In those languages that optionally or obligatorily conjoin elevational meanings with distance, it is almost always the distal demonstratives that express elevation, whereas medial or proximal demonstratives can lack elevational distinctions. For example, in Andi (Table 5), only the distal demonstrative roots can attach elevational suffixes. In Muna and Eipo (Tables 1, 2), elevational semantics and distal deixis are obligatorily co-expressed.

Kewapi (Enga-Kewa-Huli, Southern Highlands of Papua New Guinea) has a rich set of 13 demonstratives of which nine co-express elevational meanings, and relative distance and at the same time additional distance from the speaker (‘away from the speaker’) (Table 10; Yarapea, 2006, pp. 75–79). As Table 10 shows, the elevational demonstratives that encode relative proximity and middle distance are morphologically complex in contrast to the elevational demonstratives that encode relative distance. This can be taken as another way of the default co-expression of elevation with further distance as opposed to proximity or middle distance.

**TABLE 10:** The demonstrative system of Kewapi (Yarapea, 2006, p. 77).

	Relative distance –>	Close		Mid	Far
		Close	Mid		
	Specific location	*gó*	*go*		
	Generic location	*o*	*apo*		
Away	Upward [UP]	*sopo*		*sogo*	*só*
from	Downward [DOWN]	*nopo*		*nogo*	*nó*
speaker	Horizontal [LEVEL]	*mopo*		*mogo*	*mó*

Even in a language such as Lak, in which the elevational demonstratives cannot unambiguously be analyzed as co-expressing distance or proximity to the hearer or a third referent, they are not used when the respective locations are so close that the speaker can touch them with her/his finger (e.g., a hat on the head is not located UP).^13^ Thus, it seems that elevational demonstratives largely refer to areas outside the peripersonal sphere in a similar way as simple, non-elevational distal demonstratives (e.g., Coventry et al., 2008). I propose that this can be explained in the following way: in the proximal domain, fine grained semantic distinctions are superfluous since this area is accessible to the interlocutors who in the default case of a normal conversation are located in close proximity to each other [(see also Imai, 2003, p. 42) for a similar observation]. I also suggest that the same principle should apply to other semantic distinctions that demonstratives in some languages express such as visibility or audibility since such semantic categories are only relevant when the referent is not near to the speaker.

The only language I found so far that contradicts this otherwise robust cross-linguistic tendency is Yakkha. This language has two cognate sets of basic adverbial elevational roots, which are classified in the grammar as ‘topographic.’ The first set, which in the grammar is called ‘/u/-forms’ based on their stem vowel, is given in the lower part of Table 11. According to Schackow (2015, p. 187), the ‘/u/-forms’ combine with the proximal demonstrative (singular *na*, non-singular *kha*), but not with the distal or anaphoric demonstratives (Table 11). Thus, items such as *tunna* or *tukha* are morphologically complex, consisting of a morpheme with elevational meaning, followed by a morpheme with (originally) proximal demonstrative meaning.^14^

**TABLE 11:** The structure of Yakkha demonstratives,/u/-forms (Schackow, 2015, pp. 94, 187).

	Singular	Non-singular/non-count
Proximal	*na*	*kha*
Distal	*nna*	*ŋkha(ci)/nnakha(ci)*
Anaphoric	*honna*	*hoŋkha(ci)*
Proximal-up [UP]	*tunna*	*tukha*
Proximal-down [DOWN]	*munna*	*mukha*
Proximal-across [ACROSS]	*yunna*	*yukha*

This type of co-expression or combination of distance and elevation in demonstratives is not obligatory because there are languages such as Makalero (Table 3), Hatam, Iaai, Hua, Tidore, and Baskeet (8), in which elevational demonstratives are unmarked for distance and cannot be co-expressed with distance. However, those languages constitute a minority.

I encountered only very few cases of elevational demonstratives that combine with person-based deictic systems and therefore express person-based elevational meanings, e.g., Manambu (24) (Table 8).


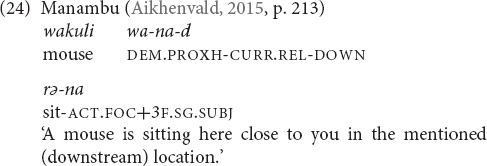


If languages have elevationals and person-based deictics, these meanings are more commonly separately expressed as, for instance, in Muna, Daga (Table 9) or Sanzhi Dargwa. The reason for the relative rareness of person-based elevational demonstratives is probably unnecessary specificity. In practice, locations above the speaker and above the addressee during a conversation largely coincide.

On the horizontal plane, the genuinely vertical dimension can, in principle, be translated into FURTHER/NEARER (or FRONT/BACK) along the sagittal axis (Bender and Beller, 2014). This means that FURTHER is equated with UP and NEARER with DOWN. This kind of projection happens at least in Sanzhi Dargwa (Forker, 2019), Tulil (Meng, 2018, p. 266), Nungon (Sarvasy, 2014, p. 413) and Belhare (Bickel, 1997), and has been called ‘person-morphic mapping’ by Bickel (1997, pp. 58–60, 68). In Sanzhi, the projection occurs not only within the local, peripersonal sphere, for example, items on a table in front of the speaker are located as UP when they are further away and DOWN when they are closer to the speaker (but always in front of the speaker). The same kind of projection is applied at the global scale on an imagined map, e.g., Estonians are located UP with respect to Latvians because Estonia is further to the north (Forker, 2019). The projection can be explained by the fact that due to their upright position human beings have to move the head downward in order to look at proximal items whereas the gaze goes upward in order to look at distal items (see Bickel (1997 and references therein). An alternative explanation could be that positions further away from the speaker are (almost) unlimited in the sense that there is no clear and unambiguous natural boundary or limit (e.g., if we climb up a mountain we can see even further away). Similarly, there is no unambiguous natural boundary or limit for the direction upward of the vertical axis. By contrast, the direction downward is limited by the ground as are locations near or close to the speaker limited by the position of the speaker.^15^

### Cardinal Directions

There are a number of languages whose elevational demonstratives also encode cardinal directions, but these meanings seem not to be available within the local domain. Examples were given in (16) and (19) from Galo. Other languages are Makalero, Bantawa, Baskeet, and Sougb. Usually only two opposite cardinal directions are encoded. Which elevational expresses which compass direction depends on the local position of the mountains that serve as anchor points and thus varies from language to language. For instance, as (25) shows, in Galo we have UP = north, DOWN = south, and LEVEL = east or west. The first two equations are also found in Bantawa. In Makalero and Baskeet, the relation is UP = east and DOWN = west, (and Baskeet has additionally ‘over there’ = north/south). In Sougb the equation is the opposite, namely UP = west and DOWN = east (26). In Iaai, the elevationals are in complementary distribution with other items that also convey compass points.


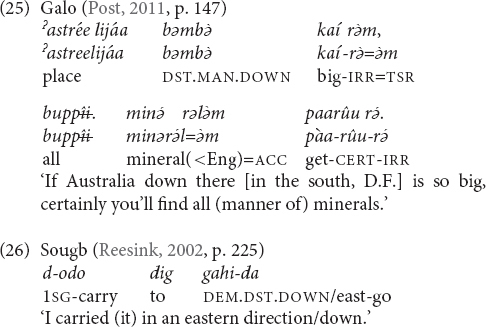


### Temporal Reference

In three languages of my sample, the UP-demonstratives carry the temporal meaning FUTURE, whereas the DOWN-demonstratives encode PAST (Tulil, Ma Manda, and Towet dialect of Nungon). The languages are spoken in Papua New Guinea, but in different areas of the country, and they belong to two different language families. In Iaai, an Oceanic language from New Caledonia, only the second equation, i.e., DOWN = PAST exists. In the following, I will provide examples from the four languages and discuss this type of spatial metaphor. I will also mention a few other languages in which spatial verticality metaphorically maps onto time.

Tulil (Taulil-Butam) has three morphologically complex demonstrative stems with elevational meaning that can be used for temporal expression (Meng, 2018, pp. 240, 263, 271). The first two demonstratives are formed by reduplication and the third one by compounding:

• *mə* ‘down, downhill, downstream’ > *pmə* ‘down distal, back’ > ‘(near/far) past.’
• *bo* ‘up, uphill, upstream’ > *pbo* ‘front, up near’ > ‘(near/far) future.’
• *mu* ‘far from speaker and hearer’ + *mə* ‘down’ > *mumə* ‘down distal’ > ‘far past/future.’

When functioning as demonstrative determiners, they can be employed with nouns such as *vənu(=a)* ‘day,’ *atade(=e)* ‘week,’ *vəgam(=e)* ‘month,’ or *laləng(=a)* ‘year,’ whereby demonstratives can precede or follow the noun (27). They are also used as independent demonstrative pronouns. The temporal meaning of the first two elevational demonstratives can be schematized as DOWN = BACK = PAST and UP = FRONT = FUTURE, and it is possible that the temporal meanings are, in fact, based on the ‘front’/‘back’ meanings. It is well known that words for ‘front’ and ‘back’ are commonly used as temporal metaphors in a wide range of different languages and cultures (e.g., Traugott, 1978; Haspelmath, 1997, pp. 56–63; Bender and Beller, 2014). I do not have an explanation for the third demonstrative and the grammar provides only one example (27), in which its meaning seems to correspond to the meaning of the first and is thus in accordance with the DOWN = BACK = PAST schema.


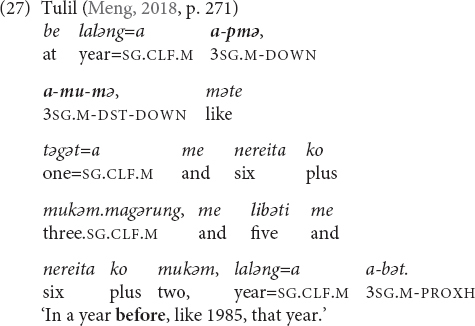


Note that in the following example the elevational morpheme is actually an adverbial demonstrative with originally spatial function (due to the locative prefix *nə*-> *nə-p-bo* ‘up there’), but it has been translated with a temporal meaning.


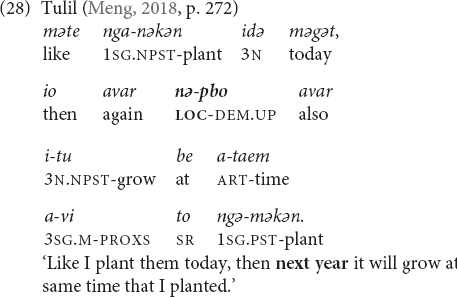


Ma Manda (Finisterre–Huon), has a three-level contrast in elevation (DOWN/UP/LEVEL), in contrast to Tulil, which has only terms for UP and DOWN, co-expressed with distance such that we arrive at six items (Pennington, 2016, pp. 287–295). The demonstratives also express temporal meanings similar to Tulil, i.e., UP = FUTURE and DOWN = PAST, and the items with the LEVEL-meaning do not cover temporal functions. Moreover, Ma Manda speakers gesture upward and downward in accordance with the meaning of the demonstratives when they refer to future and past, respectively.


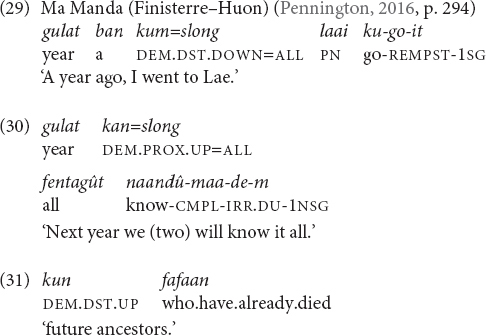


The temporal usage of the elevational demonstratives in the Towet dialect of the related language Nungon is identical to that of Ma Manda (Sarvasy, 2014, pp. 413–414).

In Iaai (Oceanic, Loyalty Islands) the deictic particle *jii* ‘down (and toward the sea)’ can express the meaning ‘past’ (32), and also serves to introduce relative clauses with past time reference. According to Ozanne-Rivierre (2004, p. 135), there are other Austronesian languages such as Taba with the same temporal extension DOWN = PAST.


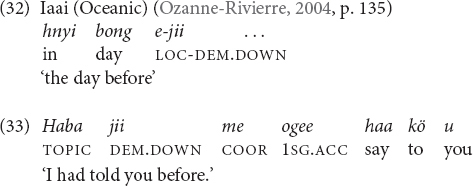


There are three other languages in my sample that do not employ their elevational demonstratives with temporal meaning, but make use of the same or a very similar type of metaphor, namely Yupno (which belongs to the same language family as Tulil), Avar, and Lak.^16^ Yupno speakers have been found to consistently use topographic (i.e., geocentrically anchored) gestures toward the ground for referring to the present, uphill for reference to the future and downhill for past (Núñez et al., 2012). The language has also one temporal expression employing a spatial metaphor *omo-ropmo bilak* (down.there.other.side year) ‘a couple years ago, a few years ago.’ In Avar, the adverbials *ʁorƛisa* ‘last year’ and *t’adejaɬːu* ‘next year’ originate from the adverbs *ʁorƛ* ‘down(ward), under’ and *t’ade* ‘up(ward),’ respectively, and in Lak *jalunè’in* ‘next year’ is derived from *jalu(w)* ‘up(ward).’ Finally, in Tzeltal, which does not have elevational demonstratives, the topographic terms -*ajk’ol* ‘uphill’ and -*anil*/*alan* ‘downhill’ are also employed with the meanings ‘later’ and ‘ahead of time, before.’ Brown (2012, p. 10) analyzes those expressions as providing evidence for the metaphor ‘time moves uphill’ or ‘the future is up(hill).’

I take the examples (27)–(33) as metaphors that map spatial expressions onto a temporal dimension: the future is located above or higher than the deictic center, and the past below. The metaphor can be explained by the direction of the biological growing process of upright human beings in the course of time. During the first years of their life human beings become taller as they get older, which means that if we compare one and the same person across time in the past the same person was smaller (=DOWN) whereas in the future s/he will be taller (=UP). The same applies to many other animals and plants with an upright position (e.g., trees).^17^

These findings are particularly interesting in view of the widely debated use of Mandarin Chinese spatial terms *shang* ‘upper, up, over, above’ for past events and *xia* ‘lower, down, below, under, for future events, which show the opposite metaphorical extension (e.g., Yu, 1998, pp. 110–112; Boroditsky, 2001). Yu (1998, p. 111) argues that this conceptualization can be explained if one presupposes that on the horizontal plane the sagittal FRONT (or FURTHER) corresponds to EARLIER and BACK (or NEARER) to LATER. This metaphorical correspondence is said to result from the fact that if human beings moved by crawling on the ground their head would be in front and their feet would come last. The same applies to other animals that move with legs – the head is normally in front and turned into the direction of movement. Yu adds that in Western cultures family trees are arranged in a similar fashion: the oldest (earliest) generations are placed on the treetop and the last generation on the bottom. Radden (2003) hypothesizes that the cultural importance of the Yangtze River may have also played a role: the river flows downward and any objects moving on it would be located higher at an earlier period of the journey and lower at a later period [(see also Bender and Beller, 2014, p. 369), who call this the ‘river model’ of time]. Furthermore, gravitation might be seen as providing a ‘natural direction’ to the vertical dimension, which goes again from up downward (Bender and Beller, 2014, p. 349).

The spatial metaphors for the vertical dimension mentioned so far are not the only ones attested for elevational demonstratives in my sample. In Tidore, the elevational deictic verbs *ine* ‘upward’ and *tora* ‘downward’ are used in two temporal expressions, namely *mulamula ine* ‘early morning, at sunrise’ (morning + upward) and *lobino tora* ‘early evening, shortly after the sunset prayer’ (lit. night downward). In these expressions, the demonstratives most likely refer to the path of the sun with its apparent rising and setting. In Daga, there seems to exist a correlation such that FUTURE/PAST = UP because *yampoa utu-pa* (third up.there-out.of.sight) means ‘next Wednesday’ and *wataget utu-p* (before up.there-out.of.sight) means ‘long ago’ (Murane, 1974, pp. 101–102).

To sum up, temporal uses of elevational demonstratives show once more how the mapping from space to time differs across languages and cultures. It is important to keep in mind, however, that these verbal metaphors are not necessarily indications or proofs that speakers of those languages have a vertical mental time line.

### Social Deixis, Evaluation, and Other Non-spatial Extensions

Perhaps surprisingly, it does not seem to be common to employ elevational demonstratives for the expression of social deixis, at least not in the languages surveyed for this study. So far, I encountered only two languages that are spoken in the Melanesia/West Papua area and have this type of semantic extension.

The first example comes from Tidore (North Halmahera), in which the elevational with the meaning UP is used to refer to locations and movements in the direction of the sultan’s palace even though the palace is located rather low.^18^ van Staden (2018) calls this usage ‘royal up’ and shows that in certain cases it includes *de facto* downward movement. Speakers showed some reluctance to use the ‘royal up’ when the referent was a dog because in the local Muslim culture dogs are not appreciated. She adds that there are other conventionalized usages that cannot be explained in terms of verticality or social deixis (e.g., Papua, which is located to the southeast of the island of Tidore, is referred to as UP because of sea currents and historical trading routes). The correlation between the UP-elevational and the conventional position of a powerful person represents an example of the metaphor CONTROL/POWER IS UP, for which cognitive evidence has been found by psychologists and psycholinguists (Schubert, 2005; Valenzuela and Soriano, 2009).

Bril (2004, p. 120) provides another example from Nêlêmwa-Nixumwak (Oceanic), where so-called ‘directionals,’ which are regularly added to deictic or anaphoric suffixes, which, in turn, are added to pronouns or determiners to form demonstratives, can be used for respectful reference to people of a higher social status. Sentence (34) is the only example that she cites for this use and it shows the elevational UP-directional *da* ‘up’ (without a preceding pronoun, deictic or anaphoric suffix). Bril further writes that it is generally improper to address others by name. Directional are used instead, e.g., *hey! the man up there*.


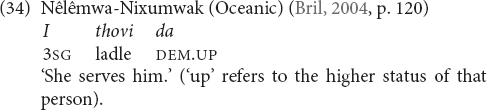


In Manambu, the noun phrases *a-da-wur du* (DEM.DST-M.SG-UP man) (Aikhenvald, 2008, p. 53) and *a-na-wur numa-də du* (DEM.DST-CURR.REL-UP big-M.SG man) (Aikhenvald, 2015) are used to refer to God (in addition to their literary sense ‘(big) man up there.’

### Elevationals as Parts of Rich Demonstrative Systems

Demonstrative systems that encode elevation are, in general, already larger than the more common systems that express only (person-based) distance. A number of languages in my sample have not only elevational demonstratives but some more terms.^19^ Other semantic distinctions with which elevational demonstratives are combined or are in complementary distribution in languages with rich demonstrative systems are

• Direction/movement: TOWARD vs. AWAY FROM^20^
  – Toward (Daga, Movima, and Lepcha).
  – Yonder/away (Ngiyambaa, Buru, Tanacross, Koyukon, and Movima).
  – Ahead (Tanacross and Koyukon).
  – Transverse (Nêlêmwa-Nixumwak).
• INWARD vs. OUTWARD (or INTERIOR vs. EXTERIOR)
  – Exterior (Jahai).
  – In/out (Central Alaskan Yupik and Eastern Canadian Inuktitut).
  – Out-of-field (Eastern Canadian Inuktitut).
  – Out in front (Dyirbal).
  - Outward (Manambu).
• Position (standing vs. non-standing) (Movima).
• Perception.
  – Invisible (Muna, Khasi, Baskeet, and Daga).
  – Visible (Daga).
  – Audible (Muna and Dyirbal).
• Other topographic meanings.
  - Seaward/landward (Iaai, Tidore, Tanacross, and Koyukon).
  - Off-river (Manambu).
• Temporary possession (Movima).
• Non-past vs. past (Movima).
• Referential or confidential (Dawro).

Most of the meanings are well-known from the literature on demonstratives (see, e.g., the lists by Diessel, 1999, p. 51; Dixon, 2003; Imai, 2003; Levinson, 2018, p. 35). Among the languages examined in this paper, Movima is particularly rich in demonstratives with unusual meanings such as ‘temporary possession’ or ‘standing position’ (Haude, 2006, pp. 177–186). Visibility has attracted some attention (Diessel, 1999, pp. 41–51; Dixon, 2003, pp. 90–91; Levinson, 2018, p. 30), but also terminological confusion (Breunesse, 2019, pp. 91–93). Levinson (2018, pp. 30, 37) suggests that in some languages invisibility might in fact better be analyzed as indirect evidentiality or simply audibility.

### Areal Distribution of Elevational Demonstratives

The examined languages come from all around the world. As explained in the Section “Materials and Methods,” the sample is a convenience sample, but based on a rather systematic and comprehensive survey of all areas of the world and more than half of the language families (Killian, unpublished). It is thus possible to suggest some generalizations concerning the areal distribution of elevational demonstratives.

There are clear areal hotspots in which there is a particular dense concentration of languages with elevational demonstratives. These areas are the New Guinea Highlands, the Himalayas, the Ethiopian Highlands and the Eastern Caucasus. Furthermore, a number of languages spoken on volcanic islands of Southeast Asia have elevational demonstratives. However, only on the island of New Guinea and immediately adjacent islands, in particular in the New Guinea Highlands, elevational demonstratives are found across a large range of different language families. In the Himalayas, only Sino-Tibetan languages have elevationals. If we consider the entire greater Hindu Kush Himalayan Region, we have to add some more Indo-Aryan languages. In the Caucasus, only East Caucasian languages, and in Ethiopian Highlands only some Omotic languages possess elevational demonstratives. By contrast, the mountainous areas of the Americas largely lack languages with elevational demonstratives with the exception of Cora and Pacaraos Quechua. The other American languages in my sample that have elevational demonstratives are spoken in rather flat areas (Movima in the Bolivian plains, Eskimo-Aleut and Na-Dené languages in Alaska and Greenland).

It has been hypothesized several times that there is a correlation between the presence of elevational demonstratives and the location of the speech community, more specifically, that the respective languages are spoken in hilly or mountainous areas (e.g., Imai, 2003, pp. 36, 38; Post, 2011, p. 152; Breunesse, 2019, p. 90; Ratliff, 2019). With respect to the languages of this paper, this claim is only partially confirmed. Five of the surveyed languages are spoken in lower hills (in general lower than 500 m above sea level), and seven languages on flat territory. All 38 remaining languages are spoken in mountainous locations mostly between 1,000 and 3,000 m (see Supplementary Appendix Table A12 for more details). This proves Holton (2019) remark that “elevation does not require mountains.” The definitions of the general elevational demonstratives given in (6) do not refer to salient landmarks. Only topographic elevational systems make a straightforward reference to mountains or hills, but as I stated above, most languages have general elevational systems and genuine topographic systems are rare. Even among the few languages which clearly have topographic elevationals, there are three languages not spoken in the mountains, but in lower hills (Dyirbal), on a flat island (Iaai) and in a flat area of Alaska (Tanacross). Holton (2019), who discusses the Eskimo-Aleut and Na-Dené languages spoken in the Artic, which is generally rather flat, notes that even though the Alaska territory includes some of the highest mountains in North America, the speakers of Na-Dené languages, which have elevational demonstrative, do not live in the mountains.

## Discussion

In this paper, I have examined elevational demonstratives, mainly focusing on their semantic and pragmatic properties. The main results of this study can be summarized as follows. The basic semantic values that elevational demonstratives encode can be ordered along a hierarchy (UP/DOWN > LEVEL/ACROSS) that reflects cross-linguistic tendencies in the frequency of the respective elevational values. Furthermore, the importance of the peripersonal sphere is linguistically reflected by elevational demonstratives because they predominantly co-express distance as opposed to proximity to the speaker. Another important finding of this study concerns the metaphorical extension of spatial elevational demonstrative meanings to the domain of time: the future is metaphorically located higher than the deictic center, and the past below. This metaphorical extension is the opposite of what has been found in Mandarin Chinese. I have proposed that the metaphor can be explained by the direction of the biological growing process of humans, many animals and plants.

In addition, I have also shown that elevational meaning *per se* is not deictic, because it does not depend on the speaker’s (or addressee’s) location, but simply relational and needs an anchor point, which can be a location that is independent of speaker or addressee. Items expressing elevational meaning can combine with deictics, in particular with demonstratives. If the combination is tight such that the items are synchronically monomorphemic, this leads to the deceptive impression that the elevational component is also deictic. Second, elevational demonstratives only rarely refer to geomorphic landmarks and they do not make use of an absolute frame of reference comparable to cardinal directions. They seem to be ‘absolute’ because normally gravity determines the direction and thus what is up and down, but the same is true for relational adverbials referring to the vertical axis.

Finally, I have argued that with respect to elevational demonstratives genealogical affiliation is more predictive than areal location. Languages with elevational demonstratives are found in flat, hilly, and mountainous regions, and they are a characteristic feature of a few language families worldwide (East Caucasian, Eskimo-Aleut, Sino-Tibetan, Timor-Alor-Pantar, Nuclear Trans New Guinea, and Omotic). New Guinea is the only area in which a wide range of languages with different genealogical affiliations that are spoken in mountain settlements have elevational demonstratives and thus geography or even language contact might have played a role in the development of those systems. In relation with that finding one possible direction for future research is to clarify whether the languages with elevational demonstratives, which were discussed in this paper, confirm the *Topographic Correspondence Hypothesis*. The latest version of the *Topographic Correspondence Hypothesis*, which is called *Sociotopographic Model*, states that languages spoken in similar topographic environments tend to have similar systems of absolute spatial reference, whereby social and cultural factors also play a role (Palmer et al., 2017). The hypothesis has been supported by data from atoll-based languages (Palmer, 2015; Palmer et al., 2017), and two languages spoken in the Hindu Kush mountain range (Heegård and Liljegren, 2018). What concerns the distribution of elevational demonstratives of the language sample used for this paper, they do not show evidence of topographical correspondence. First, there are many mountainous areas in the world without languages that have elevational demonstratives (e.g., almost all languages spoken in the American Cordillera, the Alps, the Great Dividing Range in Australia, the Atlas Mountains in North Africa, the slopes of the Great Escarpment in Southern Africa, and many more). Second, in two of the major mountain areas with elevationals, the elevationals are restricted to only one or two families. Except for East Caucasian none of the other language families spoken in the Caucasus has elevational demonstratives. In the Hindu Kush-Himalayas region, elevational demonstratives have been found so far only in Sino-Tibetan languages and a few Indo-Aryan languages (e.g., Palula, see Heegård and Liljegren, 2018 for more references). Therefore, my preliminary conclusion is to agree with Holton (2019) by suggesting that geography is less relevant than language structure and genealogy when it comes to elevational demonstratives. However, this hypothesis might obviously be rejected by new data and future studies.

There are many other open questions left for future studies of elevational demonstratives. In this paper, I have largely ignored the morphological and syntactic properties of elevational demonstratives as well as their use in discourse (e.g., as anaphors or cataphors). In order to be able to accomplish a detailed typological study we need more comprehensive descriptions of language-particular systems that are based on natural corpus data such that not only formal properties are covered but also the actual use and possibly frequency estimations can be detected. Another fruitful direction of research are various experimental approaches. The role of demonstratives in spatial cognition has been mainly investigated with respect to peripersonal space and distance as well as pointing, and the vast majority of controlled, experimental studies that I am familiar with examine languages with small demonstrative systems (English, Dutch, Italian, Spanish, Hungarian, Turkish, etc.). In the future, this line of research should be extended to languages with rich demonstrative systems such as the languages discussed in this paper.

## Data Availability Statement

All datasets generated for this study are included in the article/Supplementary Material.

## Author Contributions

The author confirms being the sole contributor of this work and has approved it for publication.

## Conflict of Interest

The author declares that the research was conducted in the absence of any commercial or financial relationships that could be construed as a potential conflict of interest. The handling editor declared a shared affiliation, though no other collaboration, with the author DF at the time of the review.

## Footnotes

1 The term ‘figure’ as used in this paper refers to the object or referent whose location is expressed and ‘ground’ is the ‘relatum’ in terms of Levinson (2003) or ground object or reference point, which can be ego or not. The third technical term employed here is ‘anchor’ or ‘anchoring point,’ which is the origin of the coordinate system by means of which the position of the figure is defined.

2 Tamil may represent an exception to this claim. According to Levinson (2003, pp. 84, 105, 108) the proximal *itu* vs. distal *atu* demonstratives also have the interpretation ‘that to the left’ vs. ‘that to the right.’

3 The relative frame of reference could be changed if the observer stands upside down on his head. In that case, object B would be located *below the head*.

4 Research in psychology has shown that the acceptability of statement that employ an intrinsic frame of reference for describing the location of a fly with respect to a donkey in a picture comparable to Figure 2 is lower than the acceptability of statements that make use of an absolute frame (Carlson-Radvansky and Irwin, 1993). See also Friederici and Levelt (1990) for similar results. Friederici and Levelt (1990) also tested two astronauts during space flight and found that if gravity is not present the relative frame of reference as determined from the own position and the head-retinal coordinates is preferred above visual background cues such as the position of trees.

5 Therefore, the study does not include items that have been characterized as deictic, but explicitly not as demonstratives as, for examples, the elevational deictic directionals analyzed by Cauchard (2018). It is of course desirable for future research to also include them and systematically compare them with elevational demonstratives (thanks to an reviewer for pointing that out to me).

6 I thank an reviewer for suggesting this classification, which differs from my original classification.

7 Only the elevational marker with the meaning UP.

8 One reviewer pointed out that the verticality values in (1) could further be subdivided into those with static meaning and those with directional meaning. The first type expresses static vertical relations and could be used in answers to the question *Where is X?* (*above/below/at the same level/across*). The second type combines the spatial relation with the expression of motion or path, has directional meaning and thus answers the question *Where is X moving to?* (*up/upward/down/downward/along the same level/across*). However, this further semantic distinction is usually not discussed in the reference grammars and descriptions that I used for this paper. The table in the Appendix provides the English translations for all items surveyed (sometimes translated from another language such as Russian). As the table shows, the majority of elevationals have been translated by ‘up’ and ‘down.’ A few descriptions use ‘above’ and ‘below,’ ‘higher’ and ‘lower,’ and some employ both static relational and directional terms. I am not in a position to judge if the translations reflect a cross-linguistic tendency for elevationals to encode more often directional than locational meanings. This question can only be answered by future research and in particular by more detailed transcriptions.

10 As one reviewer pointed out, candidate additions might be ‘up/down wind’ and ‘up/down ocean current.’ On the other hand, it needs to be clarified by further investigation whether those categories and also ‘upriver/downriver’ manifest any relation at all to elevational deixis in the sense of whether for these categories the vertical axis as it is determined by gravity plays any role at all.

11 In fact, in his earlier work Diessel (1999, p. 41) had claimed that elevational demonstratives are deictic with the speaker being the deictic center.

12 Most descriptions do not specify how the anchor point is determined. This can perhaps be taken as an argument that the elevational meaning part of demonstratives is, in fact, not deictic, but simply relational, and there is no need to explicitly specify that, as there is no need to specify that adverbs or adpositions such as *above* are relational.

13 This has been tested by the author during fieldwork in Dagestan in September 2019. It is not described in any of the grammars of Lak.

14 D. Schackow (email from May, 08, 2020) confirmed that it is the proximal demonstratives that combine with the elevationals, and not the distal ones, but that for reasons yet to be clarified the singular forms contain a double ‘n’ instead of the expected ‘n’ (e.g., *tunna* instead of the expected form *tuna*). Furthermore, Schackow writes that the function of the demonstrative roots when attached to the elevationals in Table 11 is in the first place nominalization and that the semantic component of distance is rather neutralized such that the composite form can be used for both proximal and distal reference.

15 I thank a student in the audience of the LingConLab (*Linguistic Convergence Laboratory*) seminar of the HSE Moscow on May 12, 2020, for suggesting that to me.

16 Don Killian (p.c.) drew my attention to yet another language from New Guinea that employs an elevational demonstrative for the expression of temporal meaning, namely Edolo. In this language, the phrase *salele elö alogogi* (week other over.there.LOC/week other up.across) refers to either last week or next week (Gossner, 1994, p. 87).

17 Another possible explanation for the UP = FUTURE connection, which was suggested to me by Michael Daniel, is that it is mediated by the commonly found FRONT = FUTURE link. This equation takes into account the projection of the vertical axis onto the horizontal plane. This means that what is up is at the same time in front or further and that provides the link to future, i.e., UP = FRONT/FURTHER = FUTURE. However, I do not see how a similar equation can explain DOWN = PAST.

18 For some speakers even the governor’s offices not very far from the sultan’s palace conditions the same type of usage of the ‘up’ elevational.

19 As one reviewer pointed out, it is an interesting question for future research to explore whether there is a hierarchy of co-expression of demonstrative semantics with these categories, and if there would be such a hierarchy, then we could examine where elevationals fall in.

20 The meanings that fall under TOWARD/AWAY FROM and INWARD vs. OUTWARD are conceptionally related to the basic NEAR/FAR distinction, which demonstratives in a large number of languages make (Diessel, 2005). All three distinctions are radial concepts that refer to half-axes that are radiating out from a central point (Bender and Beller, 2014, p. 345).

## Abbreviations

1: first person
2: second person
3: third person
A: most agent-like argument of a transitive verb
ABL: ablative
ABS: absolutive
ACC: accusative
ACT.FOC: action focus
ADD: additive focus
ART: article
AZR: adjectivalizer
CERT: certainty
CLF: nominal class
CMPL: completive aspect
COMP: comparative
COOR: coordinator
COP: copula
CQ: content question
CTR: contrastive
CURR.REL: current relevance
D: d-classifier
DAT: dative
DEM: demonstrative
DERIV: derivational affix
DOWN: down(ward)
DST: distal
DU dual: number
DUR: durative
DXVB: deictic verb
EMPH: emphasis
EXIS: existential
F: feminine
FUT: future
GEN: genitive
H: hearer
IMP: imperative
INCL: inclusive
INST: instrumental
IPFV: imperfective
IRR: irrealis
ITER: iterative
LOC: locative
M: masculine
MAN: manner
MIR: mirative
N: neuter
NMLZ: nominalizer
NON.FUT: non-future
NPST: non-past
NSG: non-singular
PFV: perfective
PL: plural
PN: proper name
POL: polite
PROG: progressive
PROX: proximal
PROXH: hearer-proximal
PROXS: speaker-proximal
PRS: present
PRT: particle
PST: past tense
PURP: purposive
REL: marker of relative clause
REMPST: remote past tense
REP: reported
RN.TOP: relator noun with the meaning ‘top’
S: speaker
SG: singular
SR: subordinator
SUB: subject
SUBJ: subject cross-referencing
TAG: tag particle
TOPIC: topic
TSR: temporal subordinator
UP: up(ward)
VIS: visible
VOC: vocative.
