# Supplementary material for: Elevation as a Grammatical and Semantic Category of Demonstratives
Source: Front Psychol. 2020 Jul 30;11:1712. doi: 10.3389/fpsyg.2020.01712 (PMC7406794; doi:10.3389/fpsyg.2020.01712)
Supplement: Supplementary file 1 [file Data_Sheet_1.PDF]

Table 12 (Appendix): Languages, elevational demonstrative systems and references

Abbreviations:
U UP
D DOWN
L LEVEL
A ACROSS

Part 1

**Language**	**ISO 639-3 [glottocode]**	**Family**	**Subfamily / Genus**	**Area**
Central Alaskan Yupik	esu	Eskimo-Aleut	Yupik	Alaska and Greenland
Kalaallisut (West Greenlandic)	kal	Eskimo-Aleut	Inuit	Alaska and Greenland
Eastern Canadian Inuktitut (Aivilingmiut)	ike	Eskimo-Aleut	Inuit	Alaska and Greenland
Tanacross	tcb	Na-Dené	Northern Athabaskan (Dene)	Alaska and Greenland
Koyukon	koy	Na-Dené	Northern Athabaskan (Dene)	Alaska and Greenland
Movima	mzp	isolate	isolate	Americas (excluding Alaska)
Pacaraos Quechua	qvp	Quechua	Quechua I	Americas (excluding Alaska)
El Nayar Cora	crn	Uto-Aztecan	Chorachol	Americas (excluding Alaska)
Zayse	[zays1236]	North Omotic	Northwest Ometo	Africa
Maale	mdy	North Omotic	Northwest Ometo	Africa
Baskeet (Basketo)	bst	North Omotic	Northwest Ometo	Africa
Dawuro	dwr	North Omotic	Northwest Ometo	Africa
Dime	dim	South Omotic		Africa
Avar	ava	East Caucasian	Avar	Caucasus
Andi	ani	East Caucasian	Andic	Caucasus
Lak	lbe	East Caucasian	Lak	Caucasus
Sanzhi Dargwa	[sanz1248]	East Caucasian	Dargic	Caucasus
Khasi	kha	Austroasiatic	Khasi-Palaung	Hindu Kush-Himalayas region
Bantawa	bap	Sino-Tibetan	Kiranti	Hindu Kush-Himalayas region
Yakkha	ybh	Sino-Tibetan	Kiranti	Hindu Kush-Himalayas region
Kurtöp (Kurtokha)	xkz	Sino-Tibetan	East Bodish (Bodic)	Hindu Kush-Himalayas region
Galo (and almost all other Tani languages)	adl	Sino-Tibetan	Macro-Tani	Hindu Kush-Himalayas region
Lepcha	lep	Sino-Tibetan	Lepcha	Hindu Kush-Himalayas region
Jahai (Jehai)	jhi	Austroasiatic	Aslian	Southeast Asia (excluding the Himalayas and New Guinea)
Muna	mnb	Austronesian	Malayo-Polynesian	Southeast Asia (excluding the Himalayas and New Guinea)

Buru	mhs	Austronesian	Malayo-Polynesian	Southeast Asia (excluding the Himalayas and New Guinea)
Abui	abz	Timor-Alor-Pantar	Alor-Pantar	Southeast Asia (excluding the Himalayas and New Guinea)
Blagar	beu	Timor-Alor-Pantar	Alor-Pantar	Southeast Asia (excluding the Himalayas and New Guinea)
Makalero	mkz	Timor-Alor-Pantar	Alor-Pantar	Southeast Asia (excluding the Himalayas and New Guinea)
Tidore	tvo	North Halmahera	(Papuan)	Southeast Asia (excluding the Himalayas and New Guinea)
Daga	dgz	Dagan	(Papuan)	New Guinea
Sougb	mnx	East Bird's Head	(Papuan)	New Guinea
Manambu	mle	Ndu	(Papuan)	New Guinea
Taulil (Tulil)	tuh	Taulil-Butam	(Papuan)	New Guinea
Dadibi	mps	Teberan	(Papuan)	New Guinea
Hatam	had	Hatam-Mansim	(West Papuan)	New Guinea
Fore	for	Nuclear Trans New Guinea	Kainantu-Goroka	New Guinea
Yagaria, Hua dialect	ygr [huaa1250]	Nuclear Trans New Guinea	Kainantu-Goroka	New Guinea
Usan	wnu	Nuclear Trans New Guinea	Madang	New Guinea
Tauya	tya	Nuclear Trans New Guinea	Madang	New Guinea
Nungon		Nuclear Trans New Guinea	Finisterre (Finisterre-Huon)	New Guinea
Ma Manda	skc	Nuclear Trans New Guinea	Finisterre (Finisterre-Huon)	New Guinea
Yupno (Yopno)	yut	Nuclear Trans New Guinea	Finisterre (Finisterre-Huon)	New Guinea
Eipo (Eipomek)	eip	Nuclear Trans New Guinea	Mek	New Guinea
Yale (Kosarek Yale)	kkl	Nuclear Trans New Guinea	Mek	New Guinea
Kewa (East dialect) / Kewapi	[kewa1250]	Nuclear Trans New Guinea	Enga-Kewa-Huli	New Guinea
Nêlêmwa-Nixumwak	nee	Austronesian	Oceanic, Kanak	Australia and Oceania
Iaai	iai	Austronesian	Oceanic, Loyalty Islands	Australia and Oceania
Dyirbal	dbl	Australian	Pama–Nyungan	Australia and Oceania
Ngiyambaa	wyb	Australian	Pama–Nyungan	Australia and Oceania

Part 2

Language	Location of settlements	Geography	Morphological form
Central Alaskan Yupik	southwestern Alaska (USA), which is rather flat	flat	roots
Kalaallisut (West Greenlandic)	historically spoken only in the coastal areas of the southwestern part of Greenland	flat	roots
Eastern Canadian Inuktitut (Aivilingmiut)	northwestern Hudson Bay Area (Canada)	flat	roots
Tanacross	flat alluvial plain of the Tanana Valley between the Tanana River and the Alaska Range (Alaska, USA)	flat	roots
Koyukon	along the Koyukuk and the middle Yukon River in western interior Alaska (USA), which is a flat area	flat	roots
Movima	Beni Department, Bolivia (lowlands region, central settlement at 144 m)	flat	roots
Pacaraos Quechua	spoken until the middle of the 20th century in the Lima region (Peru), Chancay valley (3,300 to 3,700 m)	mountainous	roots
El Nayar Cora	Sierra Madre Occidental, western Mexico (mountain peaks are Cerro Dolores with 2,480 m and Los Tecolotes with 2,360 m)	mountainous	suffixes and bound roots [two sets]
Zayse	Zayse highlands (Southern Ethiopia), around two lakes that are located higher than 1,100 m	mountainous	roots
Maale	the Maale area (Southern Ethiopia) is at an altitude ranging from about 1,000 m to 2,800 m	mountainous	roots
Baskeet (Basketo)	Gamo-Gofa Zone (Southern Ethiopia, 800 to 2,200 m)	mountainous	roots
Dawuro	Dawuro Zone (Southern Ethiopia, around 1,300 m)	mountainous	roots
Dime	former North Omo Administrative Region (Southern Ethiopia; highest elevation is Mt. Smith, 2,533 m)	mountainous	roots and bound roots [two sets]
Avar	Central Dagestan (Russian Federation), between 500 m (in the south) and 1,500 m above sea level	mountainous	roots
Andi	Western Dagestan (Russian Federation), around 2,000 m above sea level	mountainous	roots
Lak	Central Dagestan (Russian Federation), around 1,500 m above sea level	mountainous	roots
Sanzhi Dargwa	Central Dagestan (Russian Federation), around 1,500 m above sea level	mountainous	roots
Khasi	Khasi Hills (highest peak is Lum Shyllong which is 1,968 meters) and Jaintia Hills region of Meghalaya (India)	mountainous	roots
Bantawa	Bhojpur district, Eastern Nepal (around 1,600 m)	mountainous	roots
Yakkha	Eastern Nepal, village of Tumok (around 1,500 m) above sea level; villages generally spread over several hundred meters of altitude, because of fields located between the houses	mountainous	roots
Kurtöp (Kurtokha)	Northeastern Bhutan, at 1,200 to 2,600 m	mountainous	roots
Galo (and almost all other Tani languages)	area from central Arunachal Pradesh within the Eastern Himalaya (North East India, 5,000 to	mountainous	roots

	6,000 m) down to the Brahmaputra floodplain (200–300 m)		
Lepcha	West Bengal in India and South-Western Butan (greater Himalayan region; 1,000 m and higher)	mountainous	suffixes and bound roots
Jahai (Jehai)	rainforests of the Malay Peninsula (Northern Malaysia and Southern Thailand; with mountains up to about 2,180 m)	mountainous	roots
Muna	Muna (Indonesia) is a low-lying coral island (reef limestone) with hills (200 m - 400 m)	lower hills	roots
Buru	main settlements on Buru island (Moluccas of Indonesia), which is mostly mountainous (the highest peak is Mount Kapalatmada, 2,428 m), but not all villages are in the mountains	mountainous	roots
Abui	central area of Alor island (Indonesia), which is of volcanic origin and has very rugged terrain (highest elevation is 1,423 m); the term ‘Abui’ means ‘mountains’ or alternatively ‘enclosed place’	mountainous	roots
Blagar	most settlements on the volcanic island of Pura (over 1,000 m), Indonesia	mountainous	roots
Makalero	East Timor (island; highest elevation is Mount Naunuli with almost 900 m)	mountainous	roots
Tidore	most settlements on the island of Tidore (Moluccas of Indonesia), which is dominated by a large volcano (1,730 m)	mountainous	roots
Daga	Owen Stanley Mountains of Papua New Guinea (highest peak is around 4,000 m)	mountainous	roots
Sougb	eastern part of the Bird's Head Peninsula (mountain ranges up to almost 3,000 m) of Irian Jaya, Indonesia	mountainous	roots
Manambu	East Sepik Province of Papua New Guinea, most settlements on the Sepik River	lower hills	suffixes
Taulil (Tulil)	East New Britain Province on the island of New Britain (Papua New Guinea)	lower hills	roots
Dadibi	mountainous area in the Southern Highlands Province of Papua New Guinea	mountainous	roots
Hatam	Arfak mountains, Bird's Head area of Irian Jaya, Indonesia (Mount Arfak is 2,955 m)	mountainous	roots
Fore	Goroka District of Eastern Highlands Province (Papua New Guinea; elevations higher than 3,500 m)	mountainous	roots
Yagaria, Hua dialect	Eastern Highlands Province, Papua New Guinea; highest mountain in the vicinity is Mt. Michael (3,750m)	mountainous	roots
Usan	southern slopes of the Adelbert Range in the Madang province (Papua New Guinea; highest elevation is 1,716 m)	mountainous	roots
Tauya	base of the Bismark Range (up to around 1,500 m) in the Upper Ramu Valley, Madang Province (Papua New Guinea)	mountainous	bound roots
Nungon	vast rainforest holdings, which reach 4,000 m, on the Huon Peninsula, Morobe province of eastern Papua New Guinea	mountainous	roots

Ma Manda	mountainous area on the Huon Peninsula, Morobe province of eastern Papua New Guinea (1,000-1,500m)	mountainous	roots
Yupno (Yopno)	Huon Peninsula, Morobe province of eastern Papua New Guinea; village at 2,200m	mountainous	roots
Eipo (Eipomek)	eastern highlands of Eipumek District, West Papua (Indonesia); settlements and gardens at 1,300 to 2,000m	mountainous	bound roots
Yale (Kosarek Yale)	eastern highlands of Eipumek District, West Papua (Indonesia); settlements and gardens at 1,300 to 2,000m	mountainous	roots
Kewa (East dialect) / Kewapi	mountainous area in the Southern Highlands Province of Papua New Guinea	mountainous	roots
Nêlêmwa-Nixumwak	Northern New Caledonia, which is divided in length by a central mountain range whose highest peaks are Mont Panié (1,629 m)	mountainous	suffixes
Iaai	Ouvéa Island (New Caledonia), consists of a flat coral atoll	flat	roots
Dyirbal	upper Murray river of the Atherton Tableland, which is part of the larger East Australian Cordillera (Australia)	lower hills	suffixes
Ngiyambaa	New South Wales (Australia), rather flat territory	lower hills	suffixes

Part 3

**Language**	**Translations of the elevational meanings of the demonstratives**	**Elevational values**	**Reference(s)**
Central Alaskan Yupik	down, up, level	U/D/L	Holton (2018, 2019); Miyaoka (2012, pp. 345–381)
Kalaallisut (West Greenlandic)	down, up, level (Holton, 2019); above/below/inside/outside/ up coast or down coast, etc. (Fortescue, 1984)	U/D/L	Fortescue (1984, pp. 259–263); terminology from Holton (2018)
Eastern Canadian Inuktitut (Aivilingmiut)	horizontal /over; up /vertical-superior; down /vertical-inferior	U/D/L	Denny (1982)
Tanacross	above, below	U/D/A	Holton (2000, 2018, 2019)
Koyukon	up above, down below	U/D/A	Holton (2018, 2019); Leer (1989)
Movima	elevated (no contact with the ground)	U	Haude (2006, pp. 145, 177–186)
Pacaraos Quechua	same level, lower, higher	U/D/L	Adelaar (2019)
El Nayar Cora	in/along slope/not on top; foot of slope; head of slope/up on top	U/D/(L) [general] + U/D/A [topographic]	Casad (1982; 1984, pp. 252–262; 1985)
Zayse	higher, lower	U/D	Hayward (1990, pp. 273–275)
Maale	up, down, up there, down there, on level ground	U/D/L	Amha (2001, pp. 138–141); Treis (2019)
Baskeet (Basketo)	up, down	U/D	Treis (2019)
Dawuro	upwardly located, downwardly located, horizontally located	U/D/L	Amha (2012); Woldemariam (2001)
Dime	higher, lower	U/D	Seyoum (2008, pp. 74–77)
Avar	higher, lower	U/D	Charachidzé (1981, pp. 77–79)
Andi	higher, lower, same level	U/D/L	Saidova (2000, p. 282)

Lak	higher, (lower)	U/(D)	Zhirkov (1955, p. 71); Friedman (1994)
Sanzhi Dargwa	higher, lower	U/D	Forker (2019)
Khasi	up, down	U/D	Diessel (1999, p. 43); Rabel (1961, p. 67)
Bantawa	low, up, level	U/D/L	Doornenbal (2009, p. 95)
Yakkha	up/uphill; down/downhill; across; across beyond	U/D/A	Schakow (2015, pp. 183–203)
Kurtöp (Kurtokha)	up, down	U/D	Hyslop (2017, pp. 161–166)
Galo (and almost all other Tani languages)	upward (+north); downward (+south); same level (+ east, west, unknown direction)	U/D/L	Post (2011)
Lepcha	below, lower, down, beneath / up, higher, above	U/D	Plaisier (2007, pp. 68–72)
Jahai (Jehai)	up, down, located below speech situation (underneath, downhill, or downstream), located above speech situation (overhead, uphill, or upstream)	U/D	Burenhult (2008, 2018)
Muna	far away, higher than point of speaking or orientation	U	van den Berg (1989, pp. 89–91); van den Berg (1997)
Buru	up(ward), down(ward), upstream/toward emic center, downstream/away from emic center/far; across	U/D/A	Grimes (1991, pp. 167–173)
Abui	below, above, distal (undifferentiated, level)	U/D/(L)	Kratchovil (2007, pp. 111–118)
Blagar	high, low, level	U/D/L	Steinhauer (2014, p. 181, etc.); Steinhauer (1990)
Makalero	higher, lower, same elevation	U/D/L	Huber (2011, pp. 232–233)
Tidore	upward, downward	U/D	von Staden (2018, 2000)
Daga	high, up, higher level, low, down, lower level, same level, overhead, underneath	U/D/L	Murane (1974, pp. 38–39)
Sougb	west/up, east/down	U/D	Reesink (2002, pp. 224–226)
Manambu	up the mountain, up the river, down the mountain, down the river	U/D	Aikhenvald (2015)
Taulil (Tulil)	up, uphill, down, downhill	U/D	Meng (2018, pp. 239–274)
Dadibi	below, down	D	MacDonald (1976, p. 41); Whitby (1990)
Hatam	sloping up, vertically up, down	U1/U2/D	Reesink (1999, pp. 60–61)
Fore	up, down	U/D	Scott (1978, pp. 82–84)
Yagaria, Hua dialect	uphill, downhill/below, level	U/D/(L)	Haiman (1980, p. 258–260)
Usan	up, down, across	U/D/A	Reesink (1984, pp. 68–73)
Tauya	above speaker, below speaker	U/D	MacDonald (1990, pp. 99–103)
Nungon	lower, downhill, higher, uphill	U/D/L	Sarvasy (2014, pp. 404–419)
Ma Manda	up, down, level	U/D/L	Pennington (2016, pp. 268–314)
Yupno (Yopno)	up, down	U/D	Cooperrider et al. (2017); Núñez et al. (2012)
Eipo (Eipomek)	up, down	U/D/A	Heeschen (1982, pp. 84–88; 1998, pp. 143–148)
Yale (Kosarek Yale)	up, down	U/D/A	Heeschen (1982, pp. 89–95;)

Kewa (East dialect) / Kewapi	upward, horizontal, downward	U/D/L	Yarapea (2006, pp. 75–79)
Nêlêmwa-Nixumwak	upward, downward	U/D	Bril (2004)
Iaai	down (and towards the sea), down (near speaker); up (and inland); sunset+west+towards sea+down below; sunrise+east+inland+on high ground	U/D (L)	Ozanne-Rivierre (2004)
Dyirbal	downhill, uphill, downriver, upriver, up, down	U/D	Dixon (1972, p. 48; 2003, pp. 94–101)
Ngiyambaa	up, down	U/D	Donaldson (1980, pp. 139–143)
